# Prenatal Melatonin Therapy Enhances Postnatal Lung Development in a Mouse Model of Inflammation-Induced Preterm Birth

**DOI:** 10.3390/antiox14091094

**Published:** 2025-09-08

**Authors:** So Hee Park, Hee Young Cho, Jin Hyun Jun, Haengseok Song, Ji Yeon Lee

**Affiliations:** 1 Departments of Life Science, Graduate School, CHA University, Seongnam 13488, Republic of Korea; psh@chauniv.ac.kr; 2 Department of Obstetrics and Gynecology, Seoul National University Hospital, Seoul National University College of Medicine, Seoul 03080, Republic of Korea; hycho.md@snu.ac.kr; 3 Department of Biomedical Laboratory Science, College of Health Science, Eulji University, Seongnam 13135, Republic of Korea; junjh55@eulji.ac.kr; 4 Division of Life Science, CHA University, Pocheon 11160, Republic of Korea; 5 Department of Obstetrics and Gynecology, CHA Bundang Medical Center, CHA University School of Medicine, Seongnam 13496, Republic of Korea

**Keywords:** preterm birth, melatonin, fetal lung development, inflammation, postnatal lung

## Abstract

Inflammation-induced preterm birth (PTB) significantly impacts neonatal development, particularly due to fetal lung immaturity. The lungs undergo critical development both in utero and postnatally, and PTB disrupts this process, leading to impaired pulmonary function. Current treatments for promoting lung maturation in preterm infants have limited efficacy and safety. Melatonin, known for its potent antioxidant and anti-inflammatory properties, has shown promise in preventing PTB, but its effects on fetal and postnatal lung maturation remain unclear. This study evaluated the therapeutic efficacy of melatonin in a mouse model of intrauterine inflammation-induced PTB (IPTB). Pregnant mice (Pregnancy Day 17, [PD17]) were assigned to control, lipopolysaccharide (LPS), and LPS + melatonin groups. LPS (25 µg) was injected into the right uterine horn, with melatonin (10 mg/kg) administered intraperitoneally 30 min prior. Uterine tissues were collected at 6 and 24 h post-LPS administration for molecular and histological analyses. PTB occurred in seven out of eleven (63.6%) IPTB mice within 24 h of LPS injection, whereas melatonin significantly reduced this rate to 25% (2/8). In melatonin-treated mice, the downregulation of pro-inflammatory genes in uterine tissues, restoration of placental blood flow, increased lamellar body counts, and prevention of LPS-induced vacuolation in PD18 fetal lungs were observed. Furthermore, melatonin administration enhanced surfactant protein B expression and improved lung structure. In the offspring of IPTB mice that survived, melatonin further suppressed pro-inflammatory markers and promoted lung septal thickening at postnatal day 3. In conclusion, melatonin prevents PTB, mitigates inflammation, and supports fetal lung maturation in IPTB mice, highlighting its therapeutic potential for improving neonatal pulmonary outcomes.

## 1. Introduction

Preterm birth (PTB), defined as delivery before 37 weeks of gestation, has various causes and poses significant health risks [1,2], particularly due to immature organ development, especially the lungs. The earlier PTB occurs, the higher the risk of severe respiratory complications, as underdeveloped lungs can lead to oxygen insufficiency, triggering systemic dysfunction, including central nervous system impairments [3,4].

Among the causes of PTB, intrauterine inflammation is a major contributor [2]. Elevated expression of pro-inflammatory mediators such as cyclooxygenase (COX)-2, interleukin (IL)-6, IL-1β, tumor necrosis factor (TNF)-α, and prostaglandins (PGE2) induces uterine contraction, cervical remodeling, and fetal membrane apoptosis, ultimately leading to premature rupture of membranes and labor [1,2]. Additionally, inflammation disrupts placental blood flow through vasoconstriction and intravascular coagulation [5]. Compensatory mechanisms, such as increased iNOS activity and vasodilation, further exacerbate perfusion abnormalities [6,7], leading to placental hypoxia and the generation of reactive oxygen species (ROS), which can negatively impact fetal organ development, particularly pulmonary maturation [6,7,8,9]. Pulmonary artery dysfunction due to compromised blood flow is especially concerning as it can result in fetal lung injury and delayed lung maturation.

Despite advancements in neonatal care, pulmonary complications in premature infants remain a significant challenge, necessitating both prenatal and postnatal interventions. Postnatally, respiratory support, including oxygen therapy and mechanical ventilation, increases the risk of complications such as pneumothorax, pneumomediastinum, retinopathy of prematurity, and bronchopulmonary dysplasia. Artificial surfactant therapy can aid lung function, but in extremely preterm neonates—the highest risk group—it is often inadequate, as it may not fully compensate for the profound structural and functional lung immaturity [10]. In late preterm and term neonates, surfactants have been shown to benefit, including reduced mortality, air leaks, and the need for prolonged respiratory support [11]. Prenatally, corticosteroids (e.g., betamethasone or dexamethasone) are routinely administered between 24–34 gestational weeks to accelerate fetal lung maturation, with cohort studies demonstrating significantly lower neonatal mortality—particularly at 24–29 weeks of gestation [12]. However, efficacy remains limited in the most premature neonates (<28 weeks), and systemic risks—such as exacerbated maternal hyperglycemia requiring insulin therapy in gestational and pre-existing diabetes—have also been reported [13]. More recently, emerging therapies such as IGF-1 supplementation and N-acetylcysteine administration have been investigated in clinical studies as potential strategies to improve outcomes in preterm infants [14,15]. While these novel approaches show promise, their therapeutic application is still constrained by limited clinical evidence and methodological challenges, highlighting the need for further studies to establish their safety and efficacy.

Melatonin, a multifunctional antioxidant and anti-inflammatory molecule, offers a promising therapeutic approach for prenatal intervention. Melatonin exhibits potent antioxidant properties and efficiently scavenges free radicals [16]. Maintaining a homeostatic equilibrium between ROS and antioxidants is essential during pregnancy to ensure placental health and stability. Disruption of this balance can lead to complications such as PTB, preeclampsia, and intrauterine growth restriction [17]. Capable of crossing the placental and blood–brain barriers, melatonin modulates inflammation, ROS, and hypoxia—key drivers of PTB-associated lung immaturity [4,18,19]. Also, melatonin modulates immune responses by suppressing the activation of pro-inflammatory transcription factors such as NF-κB [20], thereby downregulating the expression of cytokines, including TNF-α, IL-1β, and IL-6, which are well-known inflammatory mediators that are markedly upregulated during PTB [21]. In addition to intrauterine inflammation, melatonin has shown protective effects in other experimental models, including hyperoxia-induced neonatal lung injury [22] and hypoxic–ischemic brain damage [23], where it reduced oxidative stress, suppressed pro-inflammatory cytokines, and preserved tissue integrity. These findings underscore the dual antioxidant and anti-inflammatory mechanisms of melatonin against both inflammation and oxygen toxicity, supporting its potential to prevent PTB-associated fetal organ damage. However, research on melatonin’s role in preventing PTB-associated lung complications and enhancing fetal lung maturation, especially in the context of intrauterine inflammation-induced PTB (IPTB), remains limited. The present study aims to investigate the therapeutic potential of melatonin in promoting fetal lung maturation in a mouse model of IPTB.

## 2. Materials and Methods

### 2.1. Animals

All animal care and treatment procedures were approved by the Institutional Animal Care and Use Committee (IACUC) of CHA University (Approval No. IACUC 230152). Pregnant ICR mice (Koatech, Ansan-si, Republic of Korea) were used for this study.

### 2.2. Establishment of Mouse Models of Intrauterine Inflammation-Induced Preterm Birth

Pregnant ICR mice obtained from Koatech were anesthetized with isoflurane, and a 1.5 cm midline incision was made in the lower abdominal wall after aseptic preparation. At Pregnancy Day 17 (PD17), mice were randomized to receive intrauterine injections of either lipopolysaccharide (LPS) (Sigma-Aldrich, St. Louis, MO, USA) to induce inflammation-induced preterm birth, or phosphate-buffered saline (PBS) as a control. For intrauterine inflammation, LPS (25 µg in 100 µL PBS) was injected into the uterine horn between the first and second gestational sacs. PTB was defined as delivery within 48 h post-injection. Melatonin (10 mg/kg) (Sigma-Aldrich) was administered intraperitoneally 30 min before LPS injection. For postnatal analyses, the number of live pups delivered in each group was recorded at birth, and subsequent evaluations were performed at matched postconceptional ages (PCA = gestational day [GD] at birth + postnatal days [PND]). Thus, pups born at GD20 (PBS) were counted and analyzed at PND1 (PCA21), while pups born at GD18 (LPS) were counted and analyzed at PND3 (PCA21), ensuring developmental equivalence across groups. In this study, PND3 was used as the reference developmental stage based on the LPS group (PCA21).

### 2.3. RNA Preparation, RT-PCR, and Real-Time RT-PCR

Total RNA was extracted from myometrium, decidua, placenta, and fetal lung tissues using TRIzol reagent (Ambion, Carlsbad, CA, USA), following the manufacturer’s instructions. For cDNA synthesis, RNA (1 µg) was subjected to reverse transcription (RT) using M-MLV reverse transcriptase (Promega, Madison, WI, USA) with random primers and oligo dT. The synthesized cDNA was used for polymerase chain reaction (PCR) with gene-specific primers under optimized thermal cycling conditions and annealing temperatures, followed by electrophoresis of the PCR products. DNA fragments were separated by 2% agarose gel electrophoresis in TBE buffer. Real-time RT-PCR was performed using SYBR Green Dye (Bio-Rad, Waltham, MA, USA) to quantify gene expression. A standard curve of cycle thresholds from serial dilutions of a cDNA sample was generated to determine relative transcript levels, normalized to ribosomal protein L7 (*rPL7*) cDNA. All PCRs were performed in duplicate. All raw data values from the real-time RT-PCR experiments are available in the Supplementary Excel File (Supplementary Data S1).

### 2.4. Western Blot Analysis

Uterine and fetal lung tissues were homogenized using a Polytron homogenizer (Brinkmann, Westbury, NY, USA). Proteins were extracted using PRO-PREP Protein Extraction Solution (iNtRON Biotechnology, Seongnam, Republic of Korea) containing a 1× phosphatase inhibitor (Roche Applied Sciences, Indianapolis, IN, USA). Extracted proteins were separated by sodium dodecyl sulfate-polyacrylamide gel electrophoresis and transferred to a nitrocellulose membrane (Bio-Rad). For Western blot analyses, the membranes were incubated with primary antibodies, followed by horseradish peroxidase-conjugated goat anti-rabbit or anti-mouse secondary antibodies (Invitrogen, Carlsbad, CA, USA). Immunoreactive bands were detected using the enhanced chemiluminescence (ECL) Western blotting substrate kit (Cytiva, Marlborough, MA, USA) and visualized using the ChemiDoc XRS + System (Bio-Rad) with Image Lab software v6.1.

### 2.5. In Vivo Imaging of Placental Blood Flow

To assess placental blood flow in pregnant mice, indocyanine green (ICG; 0.025 mg/100 µL, Cayman Chemical, Ann Arbor, MI, USA) was intravenously injected via the tail vein at PD17. Fluorescence imaging was performed for 30 min under anesthesia using the Pearl Impulse Imager (LI-COR Biosciences, Lincoln, NE, USA). In the LPS-treated group, the maximum placental fluorescence was consistently observed approximately 5 min after injection; therefore, representative images were selected at the time of injection (10 s) and 5 min (300 s). The “time to maximum fluorescence” was defined as the time taken for the placental fluorescence signal to reach its peak and subsequently begin to decline. Fluorescent signals in the placental region were captured, and the intensity of ICG fluorescence was quantified using Image Studio software v5.2 (LI-COR Biosciences, Lincoln, NE, USA).

### 2.6. Histochemistry and Immunofluorescence Staining

Fetal lungs were fixed at PD18 by immersing the entire pup in 4% paraformaldehyde (PFA) (Biosesang, Seongnam, Republic of Korea) prior to the onset of air breathing, whereas PND3 lungs were fixed by immersion in paraformaldehyde following cardiac perfusion with saline to remove blood. Fetuses were then immersed in 30% sucrose (Sigma-Aldrich), embedded in the optimal cutting temperature compound (Leica Microsystems, Wetzlar, Germany), and sectioned at 15 µm using a cryostat (Leica Microsystems). PND3 lungs were processed into paraffin after fixation and subsequently sectioned for histological and immunohistochemical analyses. The sections were stained with hematoxylin and eosin (H&E). For immunofluorescence studies, frozen sections were fixed in 4% PFA for 10 min, washed with PBS, and permeabilized with 0.1% Triton X-100 in PBS for 15 min at 25 °C. Non-specific staining was blocked using Protein Block Serum-Free (Dako, Santa Clara, CA, USA) for 1 h, followed by incubation with the primary antibodies against surfactant protein-B (SP-B) (1:200, ab40876, Abcam, Cambridge, UK) overnight at 4 °C. For fluorescence labeling, sections were incubated with Alexa Fluor 488-conjugated secondary antibodies (1:1000, Invitrogen) for 1 h at 25 °C. Nuclei were counterstained with propidium iodide (1:500, Thermo Fisher Scientific, Waltham, MA, USA). Slides were mounted using Gel Mount Aqueous Mounting Medium (M01, Sigma-Aldrich), and images were captured using a microscope (Carl Zeiss Meditec AG, Jena, Germany) and analyzed with the corresponding software.

### 2.7. Transmission Electron Microscopy (TEM)

Fetal lungs were fixed with 2% glutaraldehyde (Sigma-Aldrich) and 2% PFA in PBS at 4 °C for 8 h, followed by post-fixation in 1% osmium tetroxide. After washing with PBS, samples were dehydrated through a graded ethanol series (60%, 70%, 80%, 90%, 95%, and 100%) and embedded in Epon resin. Thin sections were prepared, counterstained with uranyl acetate and lead citrate, and imaged using a transmission electron microscope (H-7600; Hitachi, Tokyo, Japan).

### 2.8. Statistical Analyses

Statistical analyses were performed using GraphPad Prism (version 8.2, GraphPad Software, San Diego, CA, USA) and SPSS (version 29, IBM Corp., Armonk, NY, USA). Comparisons among three groups for continuous variables were conducted using the Kruskal–Wallis test, while comparisons between two groups were performed using the Mann–Whitney U test. Categorical variables were analyzed using the chi-square test. A *p*-value < 0.05 was considered statistically significant.

## 3. Results

### 3.1. Melatonin Prevents Preterm Birth and Reduces the Inflammatory Response

**Figure 1:**
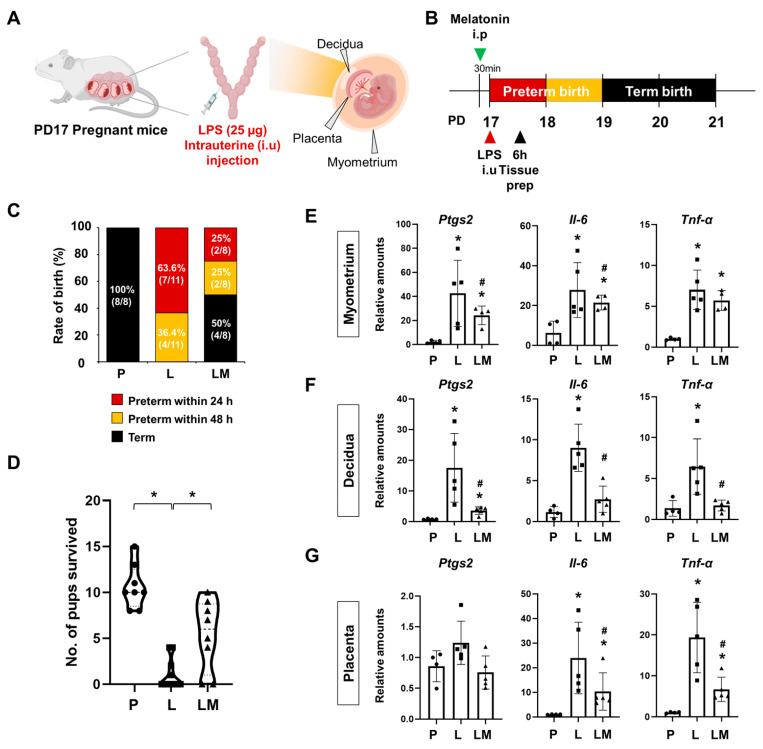
Melatonin prevents preterm birth and reduces inflammatory responses. (**A**) The scheme illustrates the establishment of IPTB mice. The figure was created with BioRender.com. (**B**) The scheme illustrates the time window defined as PTB following IPTB. (**C**) Graph showing the rate of birth after LPS administration with or without prenatal melatonin treatment. Melatonin treatment decreased the rate of PTB. (**D**) The number of surviving pups was quantified at birth. * *p* < 0.05. (**E**–**G**) Real-time RT-PCR analysis of pro-inflammatory gene expression in the myometrium (**E**), decidua (**F**), and placenta (**G**) following LPS administration, with or without melatonin treatment. *rPL7* was used as an internal loading control. Each plot represents an individual value. * P vs. others, *p* < 0.05; # L vs. LM, *p* < 0.05. Abbreviations: P, PBS; L, LPS; LM, LPS + melatonin.

To investigate the effects of melatonin on PTB, we used a mouse model of IPTB. Pregnant ICR mice received an intrauterine injection of LPS at PD17 to induce PTB, while the LPS + melatonin group was pretreated with melatonin 30 min before LPS administration (Figure 1A,B). Within 24 h, no cases of PTB were observed in the PBS group (control) (0%, 8/8). In contrast, the LPS group showed a significantly increased PTB rate of 63.6% (7/11), while co-administration of melatonin reduced the rate to 25% (2/8) (*p* < 0.05). By 48 h, PTB remained absent in the PBS group, but occurred in all cases of the LPS group (100%, 11/11) and half of the LPS with melatonin group (50%, 4/8), showing a significant difference between the groups (*p* < 0.001) (Figure 1C). In addition, we quantified the number of surviving pups after delivery (Figure 1D). The results showed a reduction in the LPS group compared with the PBS group, whereas prenatal melatonin treatment significantly increased the number of pups that survived. Melatonin treatment not only reduced the PTB rate but also significantly attenuated the expression of pro-inflammatory genes, including *Ptgs2*, *Il-6*, and *Tnf-α*, in uterine tissues such as myometrium, decidua, and placenta compared to the LPS group (Figure 1E–G).

### 3.2. Melatonin Restores Reduced LPS-Induced Blood Flow in Inflammation-Induced Preterm Birth in Mice

**Figure 2:**
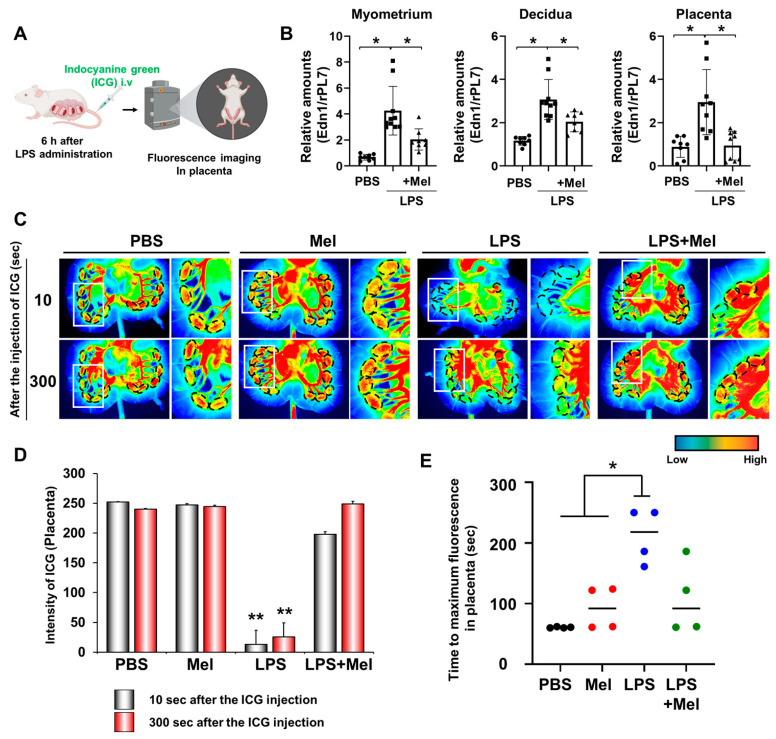
Melatonin restores reduced LPS-induced blood flow in inflammation-induced preterm birth in mice. (**A**) The scheme to explain in vivo fluorescence imaging in placental blood flow. The figure was created with BioRender.com. (**B**) Real-time RT-PCR analyses for relative mRNA expression of *Edn1* in the myometrium, decidua, and placenta. *rPL7* was used as an internal loading control. Each plot represents an individual value. (**C**) In vivo imaging of blood flow in the placenta using the Pearl Impulse Imager. LPS treatment reduced blood flow to the placenta, which was restored by melatonin administration. Regions marked by dotted lines indicate the placental location. The right panel of each group shows a magnified view of the boxed region. (**D**) Graph showing the intensity of blood flow in the placenta at 10 and 300 s after ICG injection. (**E**) Graph showing the time to maximum ICG fluorescence in the placenta. * *p* < 0.05; ** *p* < 0.001. Abbreviations: Mel, melatonin; ICG, Indocyanine green.

Adequate placental vascularization is essential for proper fetal development. To assess the impact of melatonin on placental blood flow in IPTB, indocyanine green (ICG) was administered intravenously 6 h after LPS injection at PD17 in pregnant mice, and uterine tissues were imaged using fluorescence detection (Figure 2A). The expression of the vasoconstrictor endothelin-1 (*Edn1*) was significantly increased in the myometrium (*p* < 0.001), decidua (*p* < 0.05), and placenta (*p* < 0.05) of IPTB mice (Figure 2B). Melatonin treatment effectively reduced *Edn1* expression in the uterine tissues (*p* < 0.05). In vivo imaging revealed reduced placental blood flow in IPTB mice (Figure 2C). Dotted lines indicate the location of placentas, showing decreased fluorescence intensity following LPS administration, which was restored by melatonin treatment. Quantitative analysis of ICG fluorescence intensity in the placenta revealed a significant reduction in blood flow in the LPS group compared to both the PBS and melatonin groups at both 10 s (*p* < 0.001) and 300 s (*p* < 0.001) after ICG injection. Melatonin treatment markedly restored placental blood flow at both time points (10 s: *p* < 0.001; 300 s: *p* < 0.001) (Figure 2D). In addition, the time required for placental fluorescence to decline after reaching its highest intensity was defined as the “Time to maximum fluorescence”. This time was significantly longer in the LPS group compared with the PBS and melatonin groups (*p* < 0.05), indicating impaired placental hemodynamics. However, in IPTB mice treated with melatonin, the time to maximum fluorescence did not differ significantly from that of the PBS or melatonin groups alone, but it tended to decrease, suggesting a potential partial improvement in placental blood flow.

### 3.3. Melatonin Improves Fetal Lung Immaturity in Inflammation-Induced Preterm Birth in Mice

**Figure 3:**
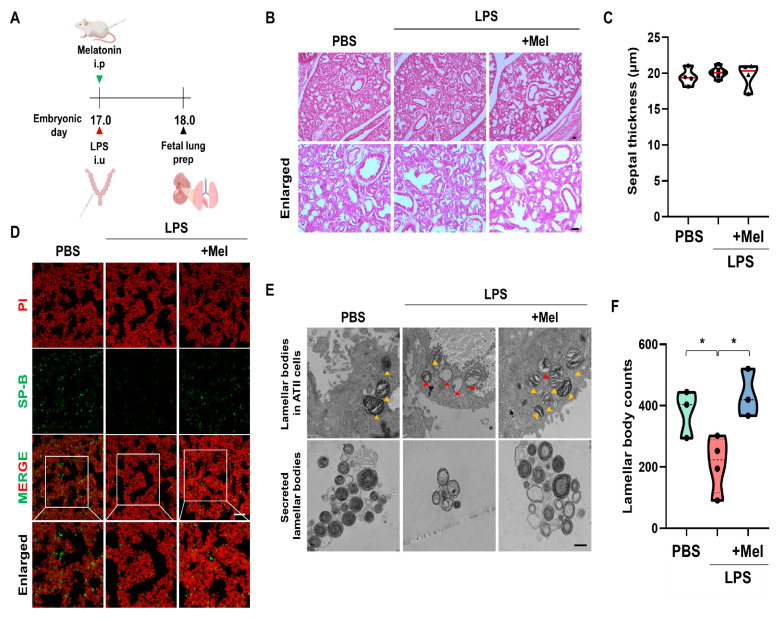
Melatonin improves fetal lung immaturity in inflammation-induced preterm birth in mice. (**A**) The scheme illustrates the fetal lung preparation time point in IPTB mice. (**B**) Histological analysis of fetal lungs at PD18 by H&E staining. (**C**) Quantitative analysis of the septal thickness in PD18 fetal lungs. (**D**) Melatonin increased SP-B expression in fetal lungs exposed to intrauterine inflammation. Scale bar = 20 µm. (**E**) Microstructure of the fetal lung by TEM. Yellow arrowheads indicate normal LBs. Red arrowheads indicate vacuolated and abnormal LBs. Scale bar = 500 nm. (**F**) The LB count in amniotic fluid 24 h after PTB * *p* < 0.05.

To assess the effect of melatonin on fetal lung development in IPTB, the number of lamellar bodies (LBs, secretory organelles present in type II alveolar cells indicative of lung development) was evaluated 24 h after LPS-induced PTB (Figure 3A). Histological analysis showed no significant structural differences in fetal lungs among the groups, regardless of LPS or melatonin treatment (Figure 3B). At PD18, septal thickness was consistent across all groups (no statistically significant difference was observed) (Figure 3C). However, LPS administration led to a decrease in SP-B expression in the fetal lungs of IPTB mice. Melatonin treatment effectively restored SP-B expression (Figure 3D). Microstructural examination by TEM showed that melatonin administration prevented vacuolation of LBs in the fetal lungs induced by LPS exposure. Additionally, melatonin increased the number of secreted LBs (Figure 3E). Furthermore, LPS treatment tended to reduce the number of LBs in the amniotic fluid (*p* < 0.05), while melatonin treatment significantly restored their numbers (*p* < 0.05) (Figure 3F). These findings suggest that although no immediate structural abnormalities were observed in fetal lungs following LPS exposure, functional impairments, particularly in surfactant production, were evident.

### 3.4. Melatonin Improves Postnatal Lung Development in Inflammation-Induced Preterm Birth in Mice

**Figure 4:**
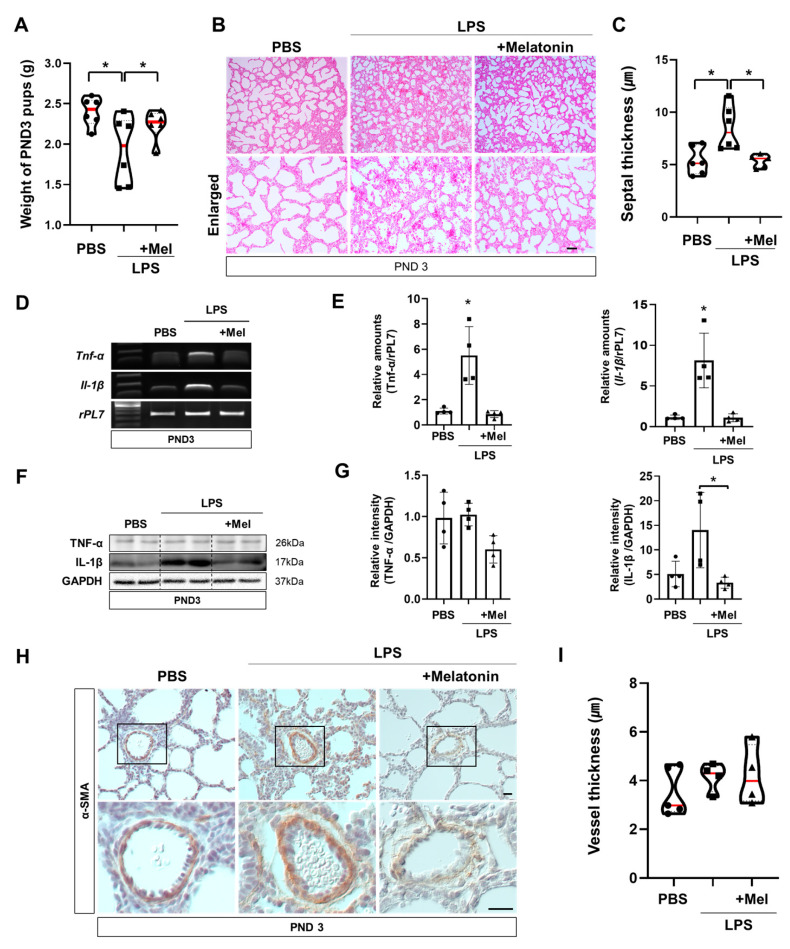
Melatonin improves postnatal lung development in inflammation-induced preterm birth in mice. (**A**) Body weights of surviving pups at PND3. PND3 was defined based on the LPS group to ensure developmental stage equivalence. (**B**) Histological analysis of PND3 lungs in IPTB mice using H&E staining. Scale bar = 20 µm. (**C**) Quantitative analysis of the septal thickness in PND3 lungs. (**D**) RT-PCR analysis of pro-inflammatory genes in PND3 lungs. (**E**) Real-time RT-PCR analysis of pro-inflammatory genes in PND3 lungs. *rPL7* is used as an internal loading control. Each plot represents an individual value. (**F**) Western blot analysis of pro-inflammatory proteins in PND3 lungs. (**G**) The relative intensity of pro-inflammatory genes was quantified using GAPDH as a loading control. Each plot represents an individual value. (**H**) Immunohistochemistry showed the expression of α-SMA in PND3 lungs. Scale bar = 20 µm. (**I**) Quantitative analysis of vessel thickness in PND3 lungs (PBS n = 5, LPS n = 5, LPS + melatonin n = 5). * L vs. others, *p* < 0.05. Abbreviations: PND, Postnatal day; α-SMA, Alpha-smooth muscle actin.

**Figure 5:**
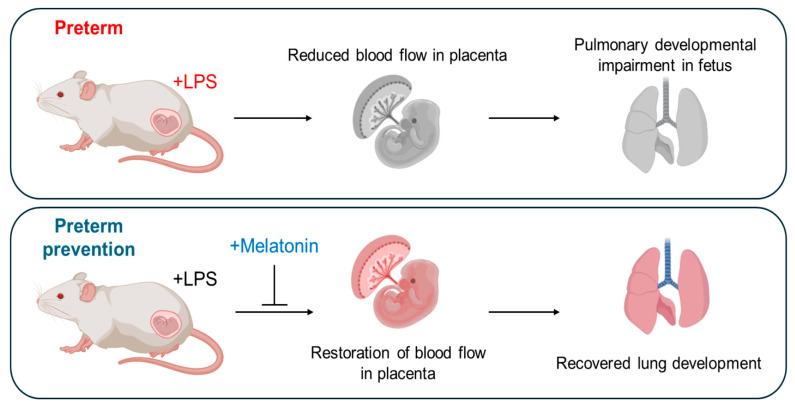
Schematic overview of the role of prenatal melatonin in recovering from preterm birth and enhancing fetal and postnatal lung development in inflammation-induced preterm birth in mice. This figure illustrates the central role of prenatal melatonin administration in regulating PTB and promoting fetal and postnatal lung development in IPTB in mice. The figure was created with BioRender.com.

Functional impairments observed immediately after LPS exposure also impacted postnatal lung development. At PND3 (PCA21), the body weights of the surviving pups were measured (Figure 4A). Offspring in the LPS group exhibited reduced body weights compared with the PBS group, whereas prenatal melatonin treatment restored the reduction. Histological examination of PND3 lungs by H&E staining revealed that the alveolar septa remained thickened in the LPS group, while this change was ameliorated in the melatonin-treated group (*p* < 0.05) (Figure 4B,C). However, melatonin administration mitigated these abnormalities (*p* < 0.01), suggesting its protective role in postnatal lung maturation. In the LPS group, *Tnf-α* and *Il-1β* were significantly upregulated compared to the PBS group (*p* < 0.05), contributing to impaired lung development at PND3. Melatonin treatment effectively alleviated these inflammatory responses (*p* < 0.05) (Figure 4D,E). Western blot analysis showed that administration of melatonin reduced the expression of inflammatory protein IL-1β compared to the LPS group (*p* < 0.05) (Figure 4F,G). Although the thickness of pulmonary capillary endothelial cells tended to increase in the LPS group, the difference did not reach statistical significance compared to the PBS and LPS with melatonin groups (Figure 4H,I). These findings demonstrate that melatonin not only restores placental blood flow to prevent IPTB but also facilitates lung maturation, mitigating alveolar septal thickness and inflammation in newborn offspring (Figure 5).

## 4. Discussion

PTB, particularly IPTB, poses significant challenges to newborn health and development, impacting crucial organ development such as the lungs. Our study provides compelling evidence for the beneficial effects of melatonin administration in a murine model of IPTB, suggesting its potential to restore fetal lung development. Using IPTB mice, our study showed that inflammation-related genes, *Ptgs2*, *Il-6*, *Tnf-α*, and *Il-1β*, were increased in uterine tissues (Figure 1) and lungs of offspring at PND3 (Figure 4). Similarly, in the chorioamnionitis-induced PTB rat model, LPS administration increased cerebral expression of inflammatory and apoptotic markers, such as IL-1β, TNF-α, RANK, and caspase-8. Similarly, in the intra-amniotic induced PTB mouse model, LPS elevated IL-6 and TNF-α expression in the amniotic fluid [24,25]. LPS exposure has been reported to induce IL-1β expression through activation of the NLRP3 inflammasome, which plays a central role in amplifying inflammatory responses [26]. The increased IL-1β mRNA and protein observed in our study suggest that this pro-inflammatory response may be mediated through NLRP3 inflammasome signaling. Consistent with this, previous studies have demonstrated that melatonin attenuates NRLP3 inflammasome activity via inhibiting nuclear translocation of NF-κB, thereby reducing IL-1β production [27,28]. These findings suggest that the reduction of IL-1β expression by melatonin in our model may be linked to its ability to inhibit NF-κB-driven priming and NLRP3 inflammasome activation. In addition, previous reports using similar PTB models have demonstrated that maternal LPS-induced inflammation not only elevates pro-inflammatory gene expression but also promotes immune cell infiltration into fetal tissues [29,30]. For example, in intraperitoneal LPS-induced PTB mouse models, marked accumulation of neutrophils was observed in fetal lungs and kidneys, coinciding with increased expression of inflammatory cytokines [29]. Also, studies in rhesus macaques have shown that intra-amniotic LPS induces a potent and rapid myeloid cell response in fetal lungs, dominated by infiltrating neutrophils and monocytes/macrophages, with transcriptional profiles indicating exposure to TLR ligands and pro-inflammatory cytokines such as IL-1β and TNF-α [30]. In addition, our unpublished observations in the uterine context revealed that LPS-induced inflammation was accompanied by increased neutrophil and macrophage infiltration, whereas prenatal melatonin treatment attenuated macrophage accumulation. Together, these observations suggest that the elevated cytokine expression in our study may reflect enhanced immune cell recruitment induced by LPS, and it is therefore reasonable to assume that prenatal melatonin treatment could attenuate such neutrophil and macrophage infiltration in the fetal lung of our model.

In our IPTB model, reduced placental blood flow led to uterine vasoconstriction, as indicated by the upregulation of vasoconstrictor markers, such as *Edn1* (Figure 2). Reduced placental blood flow impaired placental function, resulting in a hypoxic environment that initiates a cascade of events characterized by the generation of ROS and tissue inflammation [9]. Melatonin, through its potent antioxidant activity, can neutralize ROS and protect against hypoxia-induced oxidative injury, thereby mitigating the downstream effects of hypoxia in fetal tissues [17,31]. Given that melatonin administration restored placental perfusion and reduced inflammation in our model (Figure 1 and Figure 2), it is plausible that these protective effects were at least partly mediated by melatonin’s antioxidant activity.

In the chorioamnionitis-induced PTB rat model, severe hyperemia and edema were observed in various fetal organs, along with hemorrhagic foci in the pleura and lung parenchyma of the LPS group [32]. Particularly inflammatory infiltrates mainly composed of activated T lymphocytes (CD45R0+) were often associated with focal necrosis of adjacent parenchyma, exhibiting severe hypertrophy and disorganization [33]. These findings closely align with our observations of thickened fetal lung septum and inflammatory responses following intrauterine LPS administration. Notably, melatonin treatment effectively mitigated these inflammatory reactions (Figure 4). This protective effect may, in part, stem from melatonin’s ability to attenuate hypoxia-induced oxidative stress, thereby preserving vascular integrity and reducing inflammation in the fetal lung. In this study, intrauterine inflammation impaired fetal lung maturation, as demonstrated by reduced SP-B expression and abnormal LB morphology (Figure 3). These changes are consistent with the essential role of surfactant in maintaining alveolar stability and efficient gas exchange [34]. Given the essential roles of SP-B and SP-C in preventing alveolar collapse and supporting fetal lung development [35], the restoration of these proteins by melatonin underscores its potential to mitigate inflammation-induced lung dysfunction.

Melatonin exhibits potent antioxidant properties by neutralizing free radicals and acts as an anti-inflammatory agent by suppressing COX-2 and NF-kB pathways [36,37]. Clinically, melatonin has shown efficacy in improving blood flow and treating conditions such as stroke, depression, glaucoma, hypertension, diabetes, and various cancers [38,39,40]. In our previous study, melatonin prevented placental blood clot formation in a murine intrauterine inflammation model [41]. Moreover, melatonin has demonstrated anti-fibrotic potential in chemotherapy-induced lung damage [42] and ameliorates neonatal morbidities such as bronchopulmonary dysplasia by reducing oxidative stress and inflammation [41,43,44]. The antioxidant effect of melatonin is primarily mediated through non-receptor mechanisms involving direct scavenging of ROS [45]. The protective effects of melatonin in preventing PTB and improving lung immaturity are likely mediated through receptor-independent antioxidant mechanisms. Furthermore, other studies have demonstrated that melatonin’s antioxidant effects are not limited to the fetal lung but also extend to the fetal brain, where they facilitate neurodevelopment in preterm models [17,21]. These observations suggest that the antioxidant properties of melatonin may play a broader role in promoting overall fetal development under inflammatory or oxidative stress conditions. Recent research also highlights melatonin’s role in cell differentiation and proliferation [42,46,47], influencing fetal growth and development [48,49]. Our earlier investigation confirmed melatonin’s anti-inflammatory effects in preventing PTB in mice and its positive impact on fetal brain and lung maturation [6].

This study provides valuable insights into the impact of melatonin on lung development in premature infants, addressing a critical aspect of PTB. It highlights melatonin’s potential as a therapeutic option for enhancing fetal lung maturation, which could improve the prognosis of premature infants. Notably, this study extends beyond previous research focused on prenatal fetuses by demonstrating improved lung maturation in postnatal offspring. Using a mouse model of IPTB enabled a detailed investigation of the molecular pathways and physiological changes associated with fetal lung maturity under controlled experimental conditions.

However, this study has some limitations. Although it identifies changes in gene expression and histological features associated with melatonin treatment, further mechanistic studies are needed to clarify the pathways influencing fetal lung development during PTB. Additionally, the study does not assess long-term effects on respiratory function and neurodevelopment in offspring, highlighting the need for future research in this area. We monitored the offspring up to PND14, primarily recording body weights (unpublished observation). However, in the LPS group, the number of surviving pups was lower than in the other groups (Figure 1D), which reduced litter competition and consequently diminished the body weight differences compared to the other groups. This made it difficult to accurately interpret group differences at this later point. Therefore, we found our analyses on PND3, when the acute effects of intrauterine inflammation and prenatal melatonin treatment were most evident. Given its experimental nature in animals, the direct translation of these findings to clinical practice may be limited. Therefore, further investigations, including clinical trials, are necessary to validate the efficacy and safety of melatonin as a therapeutic intervention for PTB and fetal lung immaturity. Moreover, the relatively small number of biological replicates in some experimental groups could be a limitation of this study. This was largely due to the reduced survival of offspring in the LPS group, which restricted the number of samples available for certain analyses. Although statistical methods were applied appropriately, future studies with larger groups will be required to strengthen these findings. Previous studies have demonstrated that combined administration of melatonin and corticosteroids exerts greater protective effects against acute lung injury than either agent alone, suggesting potential synergistic interactions [50]. Based on these findings, we propose that future studies exploring the combination of melatonin with corticosteroids, which are the current clinical standard for promoting fetal lung maturation in PTB, may help to overcome the residual risks that remain when corticosteroids are used alone in extremely preterm infants. Such an approach could provide an enhanced therapeutic strategy to improve neonatal outcomes in the setting of IPTB.

## 5. Conclusions

This study demonstrates that melatonin effectively mitigates intrauterine inflammation induced by LPS, which adversely affects the uterine tissues, leading to preterm birth and impaired fetal lung development. The protective effects of prenatal melatonin treatment are likely mediated primarily through anti-inflammatory mechanisms that reduce inflammatory responses. They may also manifest through secondary antioxidant effects, such as the restoration of impaired blood flow. These findings suggest that melatonin holds promise as a therapeutic intervention not only for preventing PTB but also for promoting lung development in premature infants. This underscores the potential of maternal melatonin administration as a viable treatment option for complications associated with PTB. Further investigations, particularly in clinical settings, are warranted to validate the translational potential of melatonin in improving pregnancy outcomes and neonatal health.

## Data Availability

The data that support the findings of this study are available from the corresponding author upon reasonable request.

## Supplementary Materials

The following supporting information can be downloaded at: https://www.mdpi.com/article/10.3390/antiox14091094/s1, Supplementary Data S1 (Excel File): Raw data for Real-time RT-PCR and Western blot band intensity; Supplementary Figure S1: Original uncropped RT-PCR images corresponding to Figure 4D; Figure S2: Original uncropped Western blot images corresponding to Figure 4F.
