# Supplementary material for: Prenatal Melatonin Therapy Enhances Postnatal Lung Development in a Mouse Model of Inflammation-Induced Preterm Birth
Source: Antioxidants (Basel). 2025 Sep 8;14(9):1094. doi: 10.3390/antiox14091094 (PMC12466731; doi:10.3390/antiox14091094)
Supplement: Supplementary file 1 [file antioxidants-14-01094-s001.zip › antioxidants-3813599-supplementary/Supplementary figure (Supplementary Data S2).pdf]

Supplementary Figure S1. Original uncropped RT-PCR images corresponding to Figure 4D

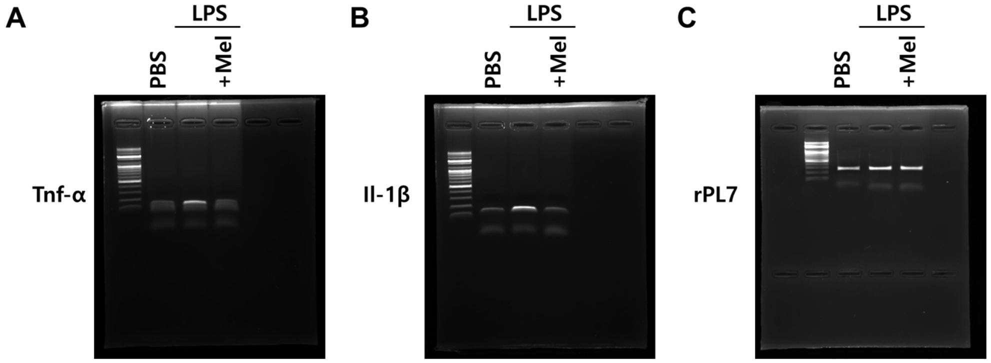

(A) Uncropped RT-PCR images corresponding to Figure 4D, showing Tnf-α. (B) Uncropped RT-PCR images corresponding to Figure 4D, showing Il-1β. (C) Uncropped RT-PCR images corresponding to Figure 4D, showing rPL7.

Supplementary Figure S2. Original uncropped Western blot images corresponding to Figure 4F

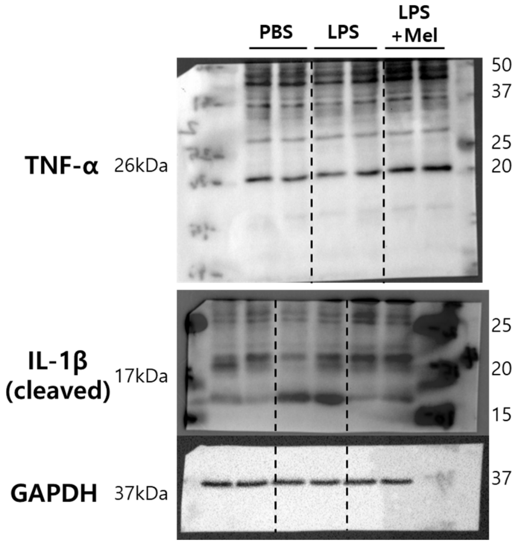

Uncropped Western blot images corresponding to Figure 4F (TNF-α, IL-1β, and GAPDH). Molecular weight markers (kDa) are indicated on the right side of each blot. For each blot, molecular weight markers were digitally merged with the corresponding blot at the time of acquisition, as provided by the imaging system. The merged images shown here therefore represent the original unprocessed data, with molecular weight markers indicated on the right side.
